# Drug repurposing for reducing the risk of cataract extraction in patients with diabetes mellitus: integration of artificial intelligence-based drug prediction and clinical corroboration

**DOI:** 10.3389/fphar.2023.1181711

**Published:** 2023-05-18

**Authors:** Zhenxiang Gao, Maria Gorenflo, David C. Kaelber, Vincent M. Monnier, Rong Xu

**Affiliations:** ^1^ Center for Artificial Intelligence in Drug Discovery, School of Medicine, Case Western Reserve University, Cleveland, OH, United States; ^2^ Cleveland Clinic Lerner College of Medicine, Case Western Reserve University, Cleveland, OH, United States; ^3^ The Center for Clinical Informatics Research and Education, The Metro Health System, Cleveland, OH, United States; ^4^ Department of Pathology and Biochemistry, School of Medicine, Case Western Reserve University, Cleveland, OH, United States

**Keywords:** aging, cataract surgery, pharmacological prevention, aspirin, acetylcysteine, ibuprofen, melatonin

## Abstract

Diabetes mellitus (DM) increases the incidence of age-related cataracts. Currently, no medication is approved or known to delay clinical cataract progression. Using a novel approach based on AI, we searched for drugs with potential cataract surgery-suppressing effects. We developed a drug discovery strategy that combines AI-based potential candidate prediction among 2650 Food and Drug Administration (FDA)-approved drugs with clinical corroboration leveraging multicenter electronic health records (EHRs) of approximately 800,000 cataract patients from the TriNetX platform. Among the top-10 AI-predicted repurposed candidate drugs, we identified three DM diagnostic ICD code groups, such as cataract patients with type 1 diabetes mellitus (T1DM), type 2 diabetes mellitus (T2DM), or hyperglycemia, and conducted retrospective cohort analyses to evaluate the efficacy of these candidate drugs in reducing the risk of cataract extraction. Aspirin, melatonin, and ibuprofen were associated with a reduced 5-, 10-, and 20-year cataract extraction risk in all types of diabetes. Acetylcysteine was associated with a reduced 5-, 10-, and 20-year cataract extraction risk in T2DM and hyperglycemia but not in T1DM patient groups. The suppressive effects of aspirin, acetylcysteine, and ibuprofen waned over time, while those of melatonin became stronger in both genders. Thus, the four repositioned drugs have the potential to delay cataract progression in both genders. All four drugs share the ability to directly or indirectly inhibit cyclooxygenase-2 (COX-2), an enzyme that is increased by multiple cataractogenic stimuli.

## Introduction

Cataract, or opacification of the lens of the eye, is a multifactorial ophthalmologic disease (Shichi, 2004). It is the leading cause of blindness in middle- and low-income countries, accounting for half of all incidences of blindness globally (Ang and Afshari, 2021). According to the National Eye Institute (NEI), cataracts affect an estimated 24.4 million U.S. citizens aged 40 or older. In addition, approximately half of all U.S. citizens aged 65 and older have suffered from cataracts (Desai et al., 1995). Diabetes mellitus (DM) is a risk factor for cataract development, as increased blood sugar levels result in cellular and biochemical damage and eventually opacification of the lens (Drinkwater et al., 2019). While the onset of cataracts in diabetes can be delayed by lifestyle modifications such as dietary changes and smoking cessation, no more than 20% of cataract surgeries are performed on DM patients (Bixler, 2019), suggesting that not all patients partake in such preventative measures. Prevention is crucial to the public health burden of cataracts, and the NEI estimates that a 50% reduction in the cataract progression rate would decrease cataract extraction by 45% (Seddon et al., 1995).

**FIGURE 1:**
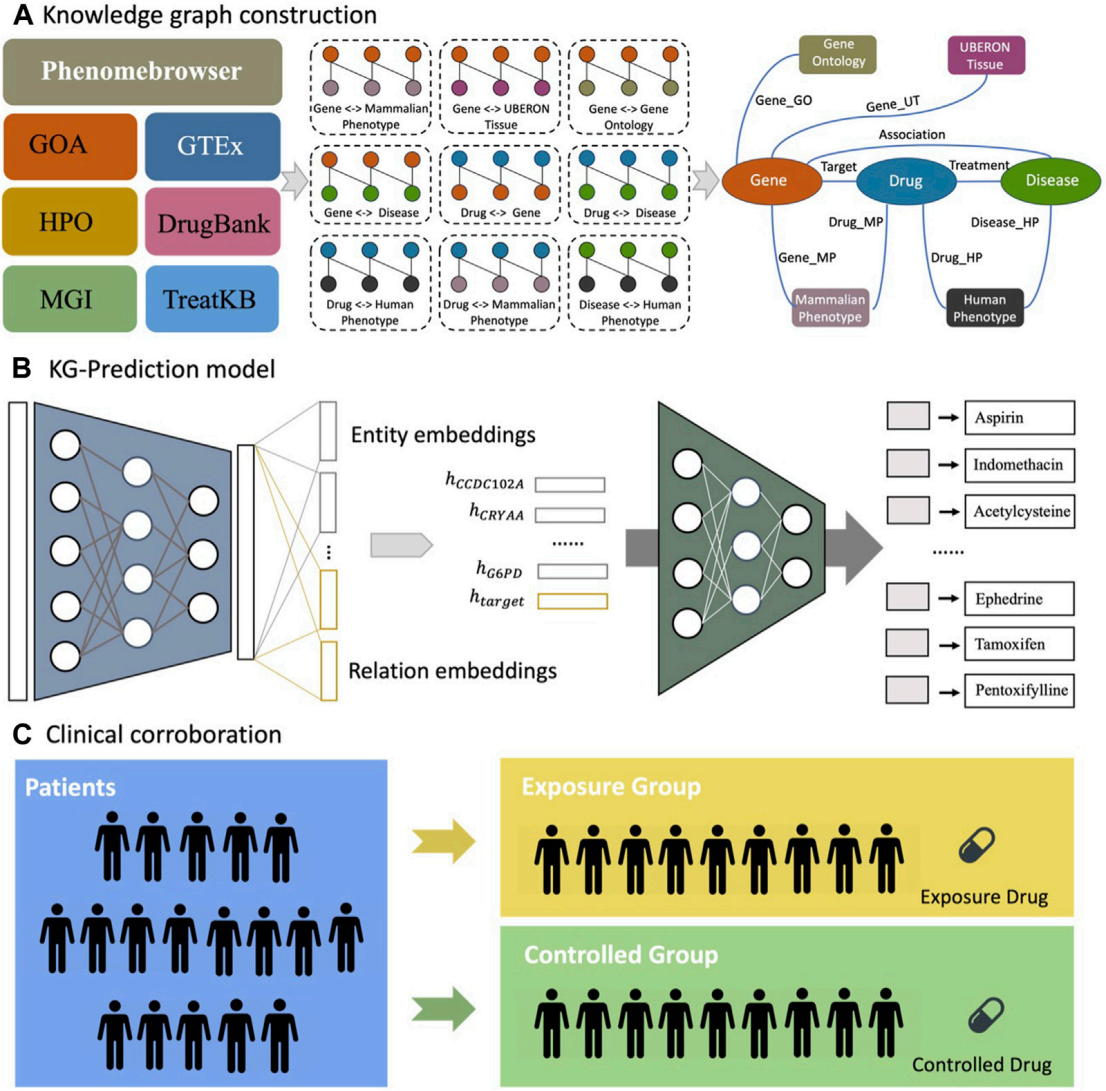
Flowchart of drug repurposing strategy to identify candidate therapies that reduce the risk of cataract extraction in DM cataract patients. **(A)** We extracted raw interactions from biomedical databases and mapped entities to standard identifiers and merged raw interactions into a knowledge graph. **(B)** The KG-Predict first modeled multi-type interactions from various biomedical databases to learn the embeddings of entities and relations. Then, KG-Predict took the embeddings of 12 DM cataract-associated genes (e.g., $h_{CCDC102A}$ ) and relation ( ${e.g.,h}_{target}$ ) as input to rank drug candidates. **(C)** Clinical evaluation of top-ranked drug candidates in reducing the risk of cataract extraction.

Currently, there are no Food and Drug Administration (FDA)-approved therapies that can prevent, delay, or cure cataracts in humans. The traditional drug discovery process for medication development is lengthy and costly (Mullard, 2014). Drug repurposing is a technique in which already approved drugs are used to treat complex diseases for which they were not initially indicated (Pushpakom et al., 2019). Numerous computational approaches have been developed to identify new treatments for various diseases (Park, 2019; Platania et al., 2020; Gesualdo et al., 2021). Recently, researchers have been increasingly using artificial intelligence (AI)-based algorithms that can analyze large amounts of chemical, genetic, genomic, and biological data to further facilitate the drug repurposing process (Levin et al., 2020; Pan et al., 2022a). Here, we developed a drug repurposing strategy (Figure 1) combining an AI-based drug discovery system with clinical corroboration using a large database of patient electronic health records (EHRs) to identify FDA-approved drug candidates for reducing the risk of cataract extraction in real-world patients with diabetes.

## Materials and methods

### Trial drug selection using the AI-based drug discovery system

We performed drug repurposing utilizing our AI-based drug discovery system termed KG-Predict, which we have recently developed (Gao et al., 2022; Gao et al., 2023) to identify potential drug candidates for DM cataracts. We first constructed the knowledge graph by extracting multiple types of interactions between drugs, genes, diseases, and phenotypic annotations from various public biomedical datasets that offered high-quality structured information (Figure 1A). Six types of phenome-level associations were collected from Gene Ontology Annotation (GOA) (Ashburner et al., 2000), Genotype-Tissue Expression (GTEx) (GTEx Consortium, 2015), Mouse Genome Informatics (MGI) (Eppig et al., 2017), and Phenomebrowser databases (OntoSIML, 2021). We also obtained two types of genome-level associations from MGI and DrugBank (Wishart et al., 2008). In our previous study, we constructed TreatKB (Xu and Wang, 2013), which included drug-disease treatment relationships mined by NLP techniques from records of patients in the FDA Adverse Event Reporting System (FAERS), FDA drug labels, MEDLINE abstracts, and clinical trial studies. To merge all interactions from different datasets into a knowledge graph, we mapped each entity to an identifier using standard biomedical terminologies. The standardized knowledge graph contained 72,360 nodes, 1,313,075 edges, seven node types, and nine semantic relationships. The statistics of entities and relations are illustrated in Supplementary Table S1.

Candidate drugs were prioritized by KG-Predict (Gao et al., 2022; Gao et al., 2023). Figure 1B provides an overview of the KG-Predict model. The KG-Predict contains an embedding module and a predicting module. The embedding module took the knowledge graph as input and learned low-dimensional embeddings of entities and relationships. Once learned, the predicting module concatenated the embeddings of entities and relations to make link predictions. For each triple (e.g., drug–target–gene), the predicting module could be represented as a ranking function that generated higher scores for true triples and lower scores for false triples. We performed target-based drug repositioning toward DM cataracts. We first obtained 12 DM cataract-associated genes from the published literature (Hashim and Zarina, 2012; Lin et al., 2013; Frankfater et al., 2020; Wu et al., 2020) based on a PubMed search under “cataract” and “diabetes” that included CCDC102A, CRYAA, KIAA1671, PPARD, AKR1B1, RPS6KA2, CACNA1C, VEGFA, VARS1, MMP2, TAC1, and G6PD. The input to KG-Predict is these genes. The output is a list of candidate drugs prioritized based on their genetic, genomic, and phenotypical relevance to diabetes and cataract, as shown in Supplementary Table S2.

### Retrospective cohort study design for top-ranked drug candidates

We clinically evaluated the top 10 drug candidates: aspirin, indomethacin, acetylcysteine, theophylline, melatonin, thalidomide, ibuprofen, ephedrine, tamoxifen, and pentoxifylline with deidentified population-level EHR data in TriNetX (TriNetX, 2023). TriNetX Analytics provides secure web-based access to patient EHR data that covers over 90 million unique patients from hospitals, primary care clinics, and specialty treatment providers. The available information on TriNetX includes demographics, diagnoses, procedures, medications, laboratory testing, vital signs, and genomic information. The platform features built-in functions that allow for cohort selection, matching incidence and prevalence analysis, and comparison of characteristics and outcomes between matched cohorts. TriNetX only provides aggregate counts and statistical summaries of deidentified patients, so no protected health information or personal data are available to its users. As a result, the Metro Health System, Institutional Review Board in Cleveland, Ohio, has determined that all research using TriNetX is not human subject research and is therefore exempt from review. We have recently used the TriNetX network platform for large-scale cohort studies (Wang et al., 2021; Wang et al., 2022a; Pan et al., 2022b; Wang et al., 2022b; Wang et al., 2022c; Wang et al., 2022d; Wang et al., 2022e).

Patients were queried in TriNetX and categorized based on their International Classification of Diseases (ICD) code and medication history. Diabetes status was based on the diagnosis of “type 1 diabetes mellitus” (ICD E10) and “type 2 diabetes mellitus” (ICD E11). Hyperglycemia status was based on the diagnosis of “elevated blood glucose level” (ICD R73). Cataract status was based on the diagnosis of “cataract in diseases classified elsewhere” (ICD H25); “other cataracts” (ICD H26); or “age-related cataracts” (ICD H28). The outcomes of interest were cataract extraction, including cataract extraction status (ICD Z98.4), cataract surgery (SNOMED 110473004), or the presence of the intraocular lens (ICD Z96.1). At the time of the study, there were 1,033,763 patients with cataracts and DM. Of these patients, 694,494 subjects had undergone cataract extraction.

**FIGURE 2:**
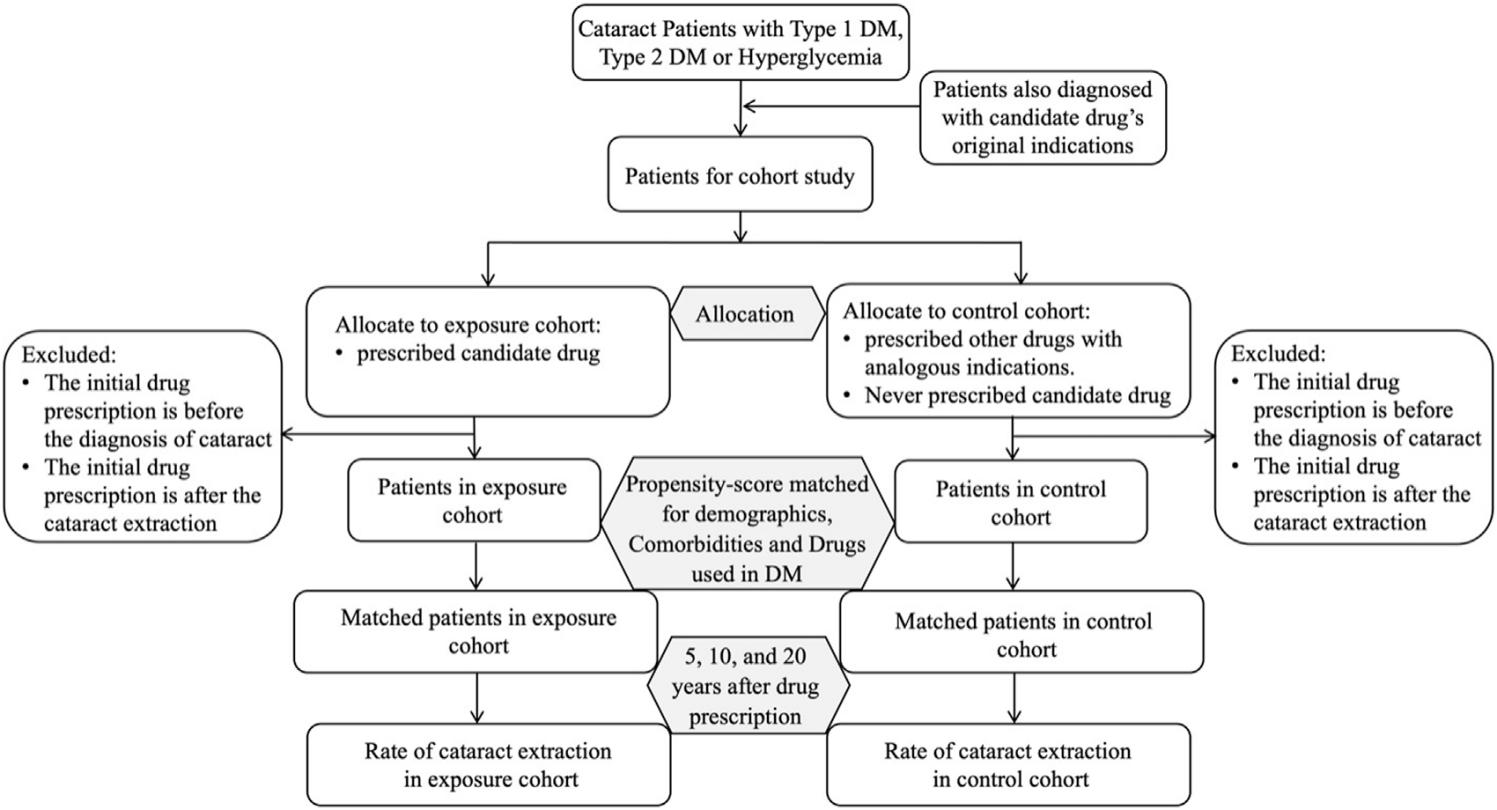
CONSORT flow diagram of the retrospective cohort study design.

We then divided cataract patients into three groups, i.e., the type 1 diabetes mellitus (T1DM) patient group, the type 2 diabetes mellitus (T2DM) patient group, and the hyperglycemia patient group, and conducted a retrospective cohort study to investigate the associations between the top 10 drug candidates and the risk of cataract extraction in each patient group. For a given drug candidate (drug A), we identified a study cohort of patients diagnosed with drug A’s original indication. The study cohort was divided into the exposure cohort and the control cohort (Figure 2). The exposure cohort comprised patients with drug A’s original indication who were prescribed drug A but never underwent cataract extraction before drug prescription. The control cohort comprised patients with drug A’s original indication who never took drug A but rather other drugs with the same indication and who also never underwent cataract extraction before drug prescription. For example, melatonin is used for treating sleep disorders, and we identified patients in the control cohort who were prescribed at least one drug belonging to the class of hypnotics and sedatives (N05C) but never took melatonin. The exposure and control cohorts were then propensity-score-matched at a ratio of 1:1 using nearest neighbor greedy matching with a caliper of 0.1 standardized mean difference (SMD) to account for potential confounding variables. The list of covariates and their standardized name codes and data types in TriNetX is described in Supplementary Table S3. These covariates include demographics (age, sex, and race/ethnicity) and known comorbidities of cataracts, such as hypertension (ICD I10–I16), glaucoma (ICD H40–H42), dry eye syndrome (ICD H04.12), degeneration of the macula and posterior pole (ICD H35.3), tobacco use (ICD Z72.0), and DM drugs (A10).

Cox proportional hazards regression was used to investigate the risk of cataract extraction at various time points after drug prescription (5, 10, and 20 years). The index event was the date of drug prescription. The rate of cataract extraction was our outcome of interest (Figure 2). The analyses were then repeated for subgroups of male and female patients.

## Results

### Associations of AI-predicted top-ranked drug candidates with risk of extraction in the three DM patient groups

**FIGURE 3:**
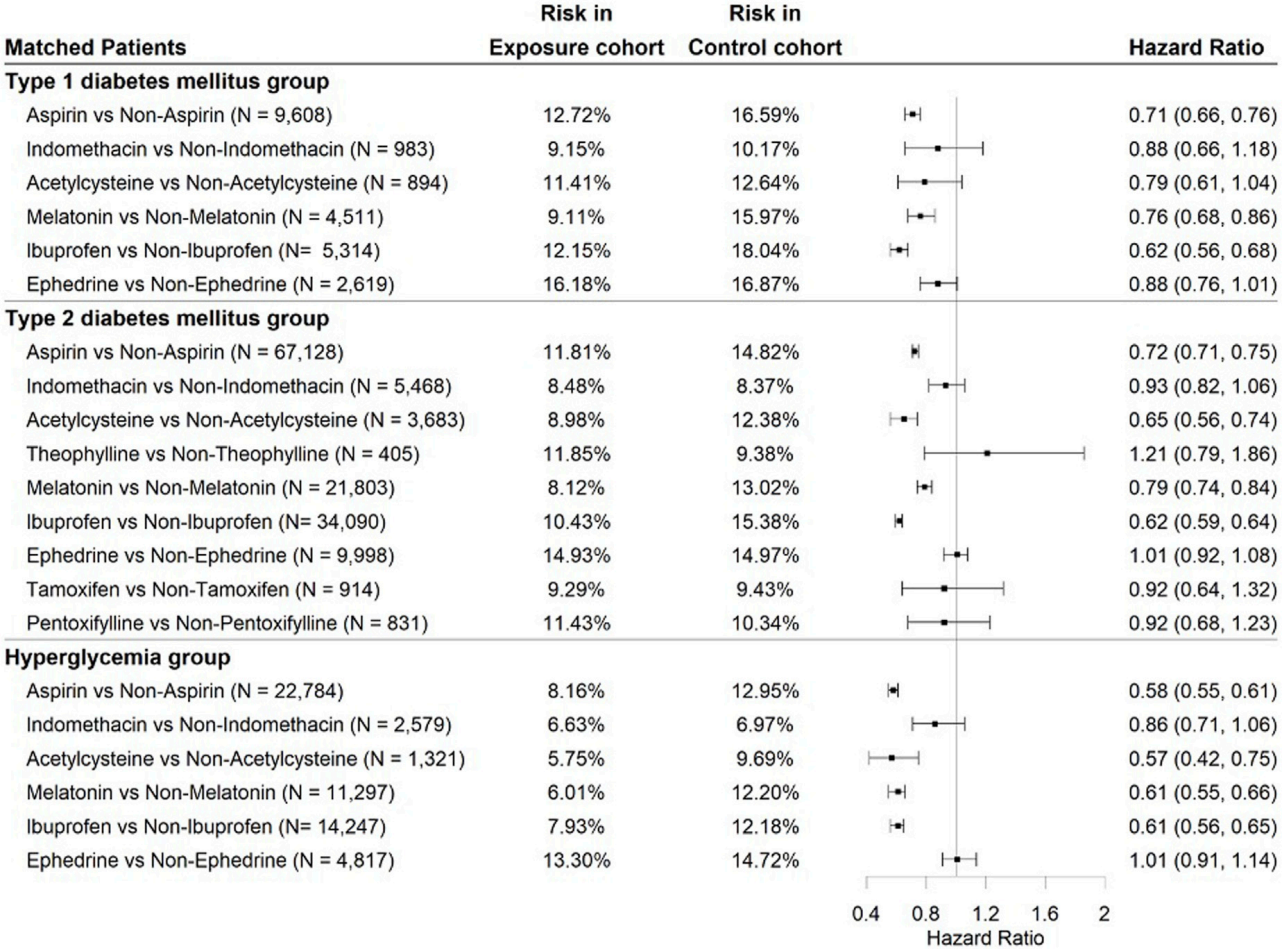
Association of top-ranked medication use with risk of cataract surgery over 5 years of the study period in the three DM patient groups using Cox proportional hazards regression. N is the number of patients in the exposure and control cohorts after propensity matching. Risk in the exposure cohort denotes the percentage of patients who underwent cataract surgery in the exposure group. Risk in the control cohort is the percentage of patients undergoing cataract surgery in the control group. The exposure and comparison cohorts were propensity score-matched for demographics, comorbidities, and medications.

We performed retrospective cohort studies to evaluate the association between the top 10 candidate drugs and cataract extraction over the 5-year study period in three DM patient groups. Figure 3 shows the hazard ratios (HRs) and corresponding 95% confidence intervals (CIs) for the risk of cataract extraction for each drug. Due to an insufficient study sample size, tamoxifen, theophylline, and pentoxifylline were excluded from the T2DM group and hyperglycemia group, and thalidomide was excluded from the three DM patient groups. The characteristics of patients who were prescribed aspirin in the T1DM patient group before and after matching are shown in Table 1. The patient characteristics for other drugs are provided in Supplementary Tables S4–S13. As evidenced in the table, matching rendered comparable exposure and control cohorts with no significant differences among covariates. Aspirin, which is used to treat pain, inflammation, and rheumatoid arthritis, was associated with a significantly lower risk of cataract extraction in the three DM patient groups, with HRs of 0.71 (95% CI: 0.66–0.76), 0.72 (95% CI: 0.71–0.75), and 0.58 (95% CI: 0.55–0.61), respectively. In addition to aspirin, two drugs (melatonin and ibuprofen) were also associated with a significant reduction in the risk of cataract extraction compared to other drugs with comparable indications in the three DM patient groups: melatonin [HR = 0.76 (0.68–0.86), 0.79 (0.74–0.84), and 0.61 (0.55–0.66)] and ibuprofen [HR = 0.62 (0.56–0.68), 0.62 (0.59–0.64), and 0.61 (0.56–0.65)]. Acetylcysteine did not meet the threshold for statistically significant associations with reduced risk of cataract extraction in the T1DM patient group but was significantly associated with decreased cataract extraction in the other two groups with HRs of 0.65 (95% CI: 0.56–0.74) and 0.57 (95% CI: 0.42–0.75), respectively. For the other five drugs (indomethacin, theophylline, ephedrine, tamoxifen, and pentoxifylline), there was no significant reduction in cataract extraction risk. Follow-up analyses were conducted for aspirin, acetylcysteine, melatonin, and ibuprofen.

**TABLE 1:** Characteristics of patients prescribed aspirin in the T1DM patient group.

Characteristic	Before matching			After matching		
	Aspirin cohort	Non-aspirin cohort	SMD	Aspirin cohort	Non-aspirin cohort	SMD
Total no.	14,376	24,180		9,608	9,608	
Age	63.6 ± 12.2	60.4 ± 13.6	0.24*	62.5 ± 12.2	62.3 ± 12.4	0.02
Sex assigned at birth, %						
Female	53.1	55.4	0.04	52.9	52.7	0.004
Male	46.8	44.5	0.04	47.1	47.2	0.004
Ethnicity, %						
Hispanic/Latinx	11.6	11.9	0.008	12.1	12.1	0.005
Non-Hispanic/Latinx	66.3	53.9	0.25*	63.5	64.6	0.02
Race, %						
African/ U.S. citizens/Black	25.4	19.3	0.14*	23.5	24.1	0.01
White	60.1	57.1	0.06	60.4	61.7	0.02
Asian	1.5	1.4	0.001	1.5	1.3	0.01
Comorbidities, %						
Hypertension	90.3	76.1	0.39*	88.5	89.1	0.01
Tobacco use	4.8	3.9	0.04	4.8	4.5	0.01
Glaucoma	26.7	11.7	0.38*	19.4	19.2	0.006
Dry eye syndrome	17.5	5.9	0.36*	10.7	10.4	0.01
Degeneration of macula and posterior pole	17.1	5.5	0.36*	10.1	9.2	0.02
Other drugs, %						
Drugs used in DM	80.4	59.6	0.46*	77.1	77.3	0.008

Note: SMD, standardized mean differences. *SMD, greater than 0.1, is a recommended threshold for declaring an imbalance.

### Association of the four candidate drugs with the risk of cataract extraction according to sex assigned at birth over different study periods

**FIGURE 4:**
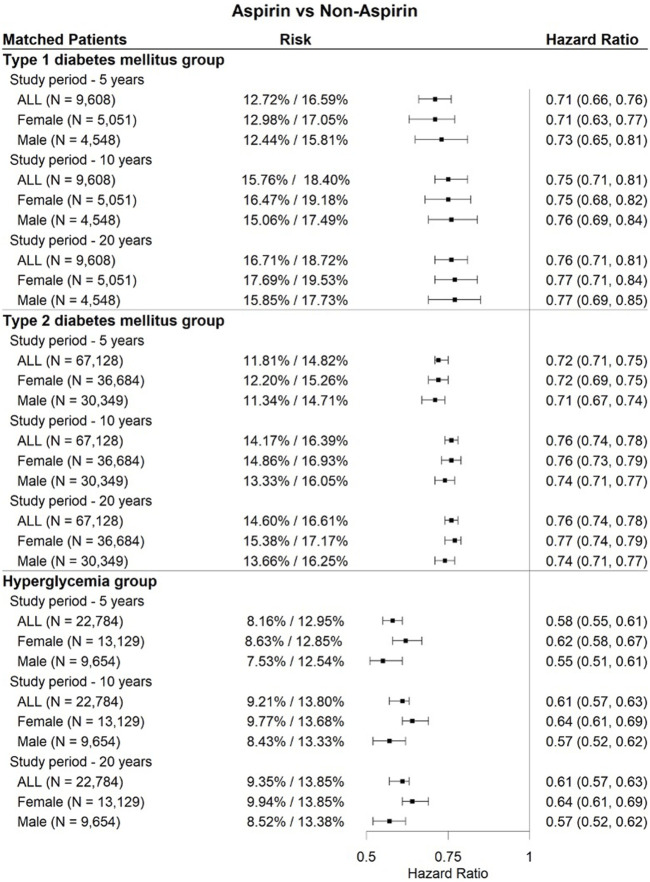
Forest plots of cataract extraction risk in DM cataract patients prescribed aspirin, divided according to sex assigned at birth, with a study period of 5–20 years.

**FIGURE 5:**
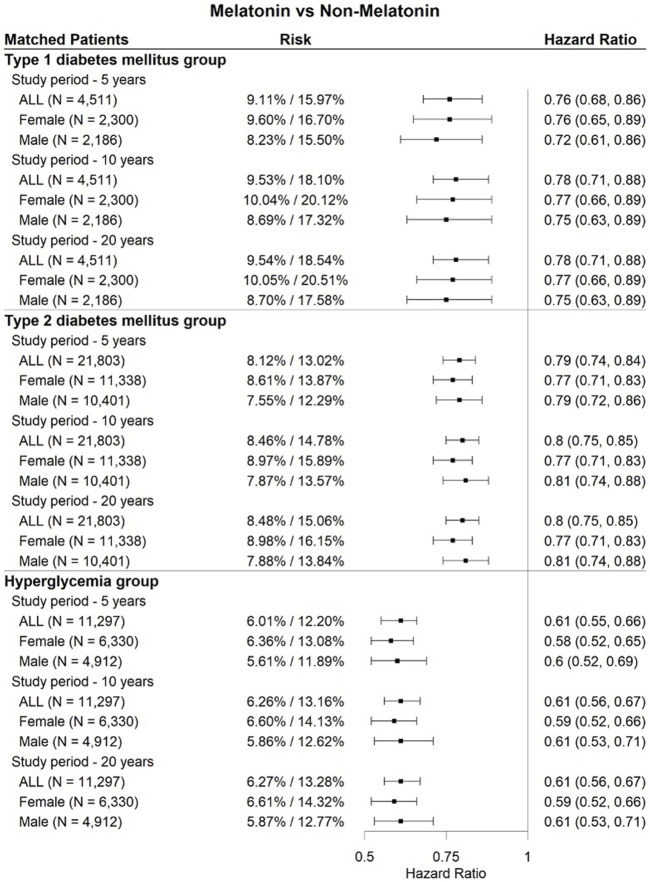
Forest plots of cataract extraction risk in DM cataract patients prescribed melatonin, divided according to sex assigned at birth, with a study period of 5–20 years.

**FIGURE 6:**
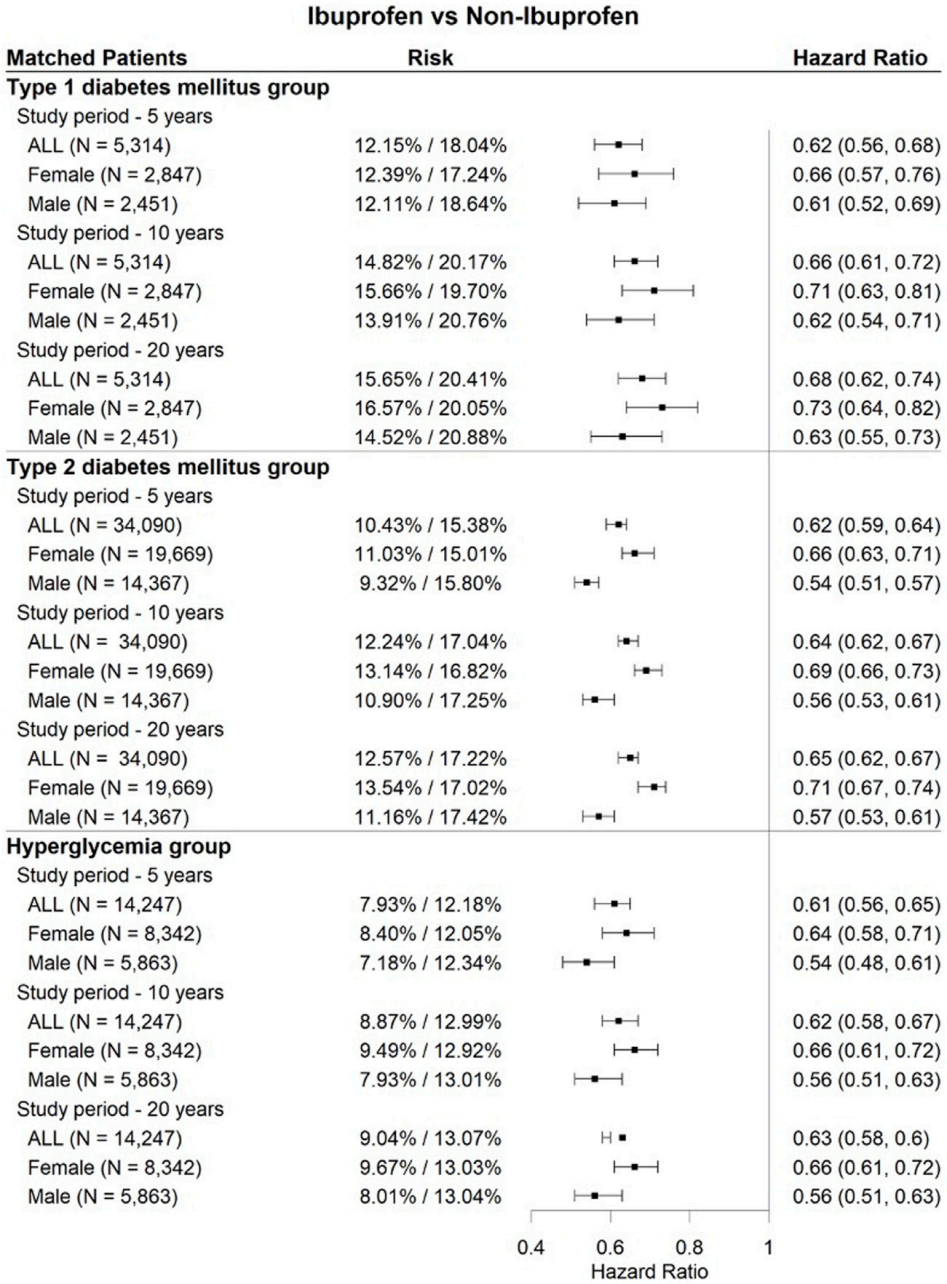
Forest plots of cataract extraction risk in DM cataract patients prescribed ibuprofen, divided according to sex assigned at birth, with a study period of 5–20 years.

**FIGURE 7:**
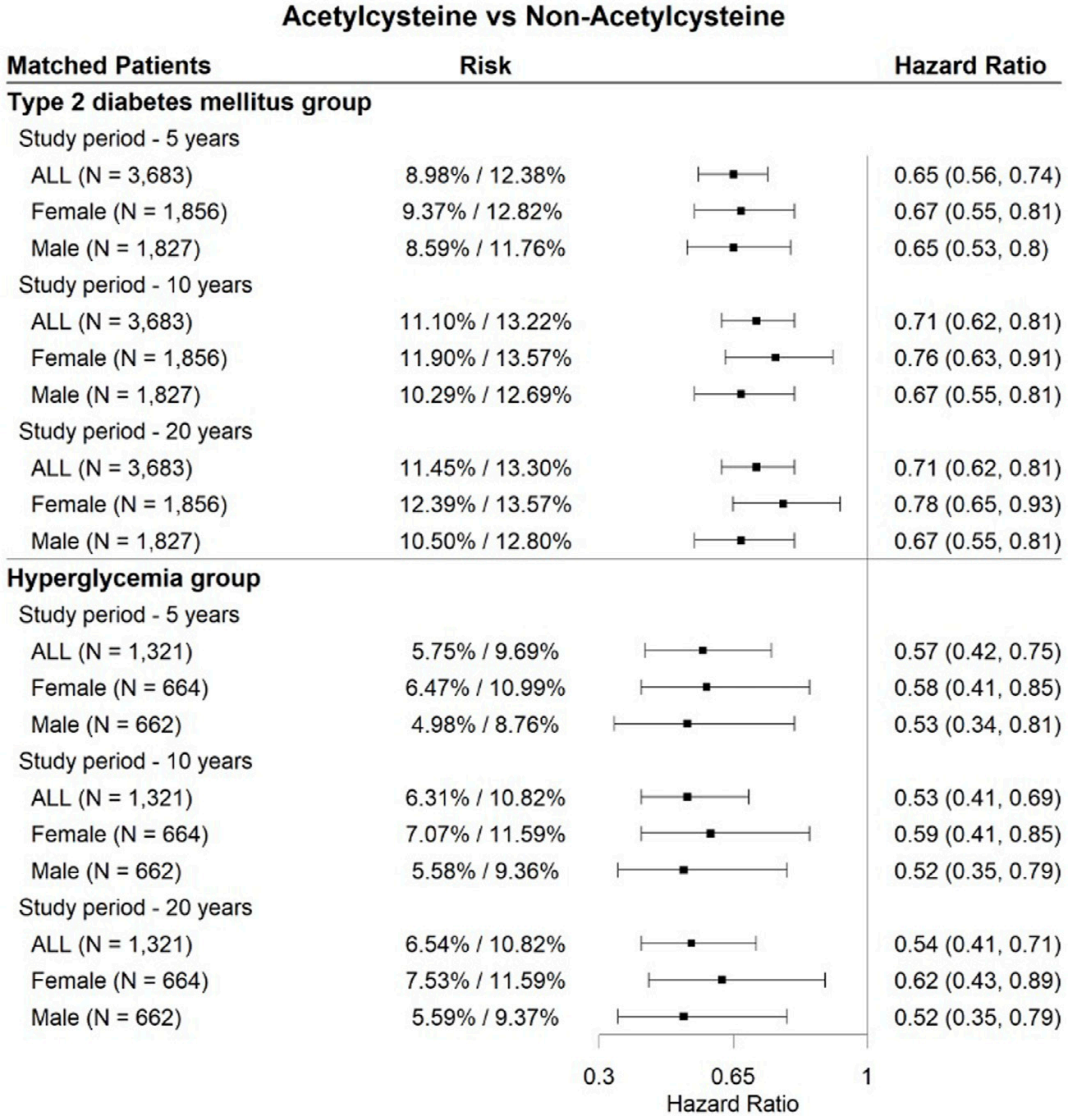
Forest plots of cataract extraction risk in DM cataract patients prescribed acetylcysteine, divided according to sex assigned at birth, with a study period of 5–20 years.

**FIGURE 8:**
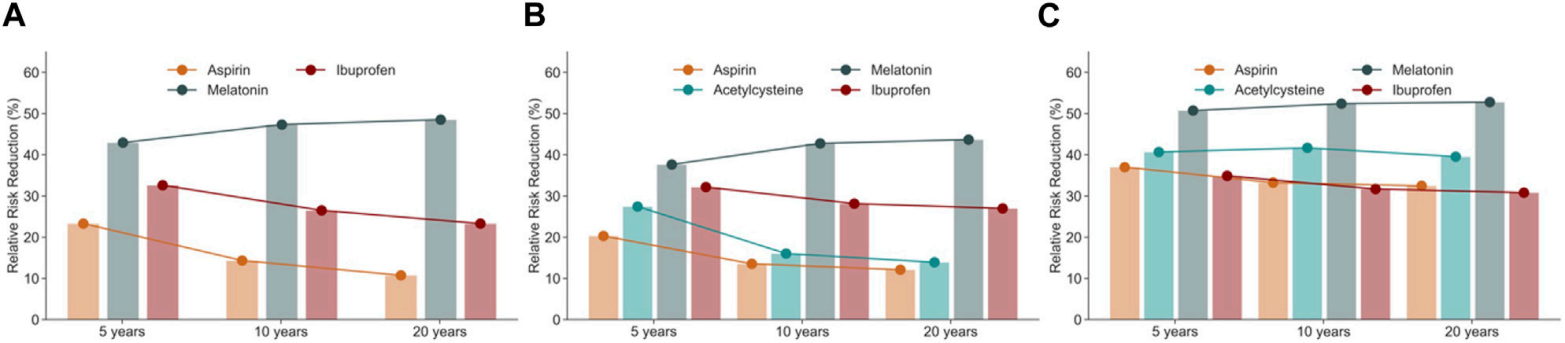
Relative risk reduction of cataract extraction in cataract patients prescribed aspirin, acetylcysteine, melatonin, and ibuprofen in three groups of diabetic patients from the TriNex database: **(A)** T1DM patient group, **(B)** T2DM patient group, and **(C)** hyperglycemia patient group. Note that acetylcysteine was not protective in the T1DM group.

As shown in Figures 4, 5, 6, three drugs (aspirin, melatonin, and ibuprofen) were associated with a significantly lower risk of cataract extraction in the three DM patient groups during a study period of 5, 10, or 20 years. Acetylcysteine displayed a significant reduction in the risk of cataract extraction compared with matched individuals who were prescribed other drugs with comparable indications in the T2DM group and hyperglycemia group during a study period of 5–20 years (Figure 7). We further investigated the effects of repurposed drugs on the risk of cataract extraction in men and women separately (Figures 4, 5, 6, 7). We observed that both male and female patients prescribed the four drugs experienced a significantly reduced risk of cataract extraction in the three DM patient groups. We finally analyzed the time trend of reduction in cataract prevalence of the four drugs in the three DM patient groups over 20 years (Figure 8). Melatonin showed a significant reduction in cataract prevalence in a sustained manner over 20 years. Patients who were prescribed aspirin, acetylcysteine, or ibuprofen also had positive effects during a study period of 5–20 years, and their effects waned after that.

## Discussion

In this study, we developed a drug repurposing strategy to identify FDA-approved drugs that delay cataract extraction in DM patients. Our approach combines an AI-based drug repurposing system with EHR-based clinical corroboration. We identified aspirin, acetylcysteine, melatonin, and ibuprofen as therapies that may be associated with decreased risk of cataract extraction in diabetic patients with cataracts.

Our study shows that patients with cataracts who were prescribed aspirin experience a lower risk of cataract extraction than matched individuals in all three DM patient groups. Findings from animal models suggest that aspirin may prevent cataract formation by reducing carbamylation (Crompton et al., 1985) of soluble lens proteins, acetylation of lens proteins, inhibition of glycation, improved glucose tolerance, and an indirect antioxidant effect (Mihail, 1990). Our results are also consistent with those of a clinical trial evaluating aspirin in DM cataract patients with rheumatoid arthritis (Cotlier, 1981), in addition to several clinical trials (Heyningen and Harding, 1986; Harding et al., 1989; Klein et al., 2001), further suggesting possible protective mechanisms of aspirin against cataract extraction. However, several other studies report no significant reduction in cataract extraction for patients taking aspirin (Klein et al., 1987; Seddon et al., 1991; Hankinson et al., 1993; Christen et al., 2001). Many such studies focus on aspirin for a wide patient population, not just those with DM, so our finding that aspirin reduces the risk of cataract extraction in DM patients with pain, inflammation, or rheumatoid arthritis should not be extrapolated to all patients with cataracts.

Acetylcysteine is used to treat dry eye syndrome and respiratory diseases. We found that cataract patients who were prescribed acetylcysteine displayed a lower risk of cataract extraction than those prescribed control drugs (mucolytics or eye lubricants) in the T2DM patient group and the hyperglycemia patient group. Acetylcysteine is an endogenously produced antioxidant that scavenges free radicals (Carey et al., 2011). There is evidence in animal models that acetylcysteine reduces cataract formation by serving as a precursor for glutathione, a potent antioxidant (Zhang et al., 2008), and through other antioxidant mechanisms (Aydin et al., 2009).

Melatonin is an endogenous hormone produced by the pineal gland that is indicated for the treatment of sleep disorders. Patients with cataracts who were prescribed melatonin displayed a significantly lower rate of cataract extraction than matched patients in all three DM patient groups. The efficacy of melatonin in preventing cataracts has already been demonstrated in animal models (Abe et al., 1994; Yağci et al., 2006; Khorsand et al., 2016). These studies suggest that melatonin’s antioxidant properties, and its reduction of lipid peroxidation and blood sugar levels, may explain the mechanism by which it prevents cataract formation (Lledó et al., 2022). However, it may also act indirectly by inducing sleep and thus act on circadian genes, either by the downregulation of deleterious, cataractogenic genes such as AKR1 (Franko et al., 2020) or by upregulating protective genes, such as Nrf2 (Xing et al., 2023).

Ibuprofen, which is an NSAID, was identified as a potential candidate drug for delaying cataract extraction. Patients with cataracts who were prescribed ibuprofen had a lower risk of cataract extraction than those prescribed other NSAIDs in all three DM patient groups. Ibuprofen inhibits the enzymes cyclooxygenase-1 and -2, reducing the synthesis of prostaglandins and functioning as a potent antioxidant (Bushra and Aslam, 2010). Several animal studies indicate that ibuprofen possesses anti-cataract activity due to its antioxidant properties (Robert and Harding, 1992) and by binding to lens proteins and preventing cross-linking events that lead to lens opacification (Plater et al., 1997).

The fact that we found that the drugs did not have additive effects in reducing the risk of cataract surgery suggests a possible common mechanism of action, with COX-2 inhibition being a shared target of all four drugs, either via direct or indirect inhibition. COX-2 levels are increased in experimental cataracts (Cao et al., 2018), and levels are increased by known cataractogenic stimuli such as hyperglycemia (Song et al., 2020), UV light (Chan et al., 2015), smoking (Huang and Chen, 2011), low glutathione levels, and oxidative stress (Robert and Harding, 1992). On the contrary, in addition to the well-known inhibition of COX-1/2 by ibuprofen and aspirin, COX-2 protein or RNA levels have been shown to be suppressed by N-acetylcysteine and other antioxidants (Cao et al., 2018; Villagarcía et al., 2018), and by melatonin via ATF6, itself a COX-2 suppressor (Bu et al., 2017).

Our study has several limitations. First, we selected 12 genes that are deemed to be highly associated with DM cataracts as our model’s input to generate a ranked list of candidate drugs. However, our knowledge of genes involved in DM cataracts is still evolving. The AI-based drug prediction system is highly dynamic and can easily incorporate new data and knowledge. Second, the EHR database has limited information on drug usage duration, dosage, and patient compliance; for example, prescriptions may be obtained from outside providers that are not recorded in TriNetX. Due to these limitations, we could not evaluate how the duration, dosage, and compliance of medication use affect the risk of cataract extraction in DM cataract patients. Third, no subgrouping of cataract types (such as cortical, nuclear, posterior subcapsular cataract, and posterior capsule opacification) was performed, as only a small percentage of EHR records contained these sub-diagnoses. Therefore, this study could not detect medications that prevent the extraction of a specific subtype of cataract. Further limitations are dependent on the accuracy of the digital data entry in TriNetX. These include drug utilization, risk factors, patient misdiagnosis, and other possible confounding factors. A potential limitation may arise from propensity score matching. Although patient matching can strictly balance the distribution of covariates between exposure and control cohorts and therefore avoid many confounders, this method cannot account for those confounders that are unobserved or unmeasured due to the observational nature of the study design, which could increase data imbalance and bias. In addition, TriNetX represents patients who had medical encounters with healthcare systems, not a random selection of individuals throughout the United States, so the conclusions drawn from our analyses may not be representative of the entire U.S. citizens population. The generalizability of the results from the TriNetX platform remains unknown and needs to be validated in other populations and all drug recipients.

In conclusion, we believe we have identified drug candidates for delaying cataract extraction in DM patients by combining a knowledge graph-based drug discovery system with clinical corroboration. We identified four drugs (aspirin, acetylcysteine, melatonin, and ibuprofen) that appear to reduce the risk of or delay cataract extraction in this patient population. Our results provide the foundation for future hypothesis-driven clinical studies of these drug candidates to further understand their efficacy.

## Data Availability

Publicly available datasets were analyzed in this study. These data can be found here: https://trinetx.com/.
