# Supplementary material for: Drug repurposing for reducing the risk of cataract extraction in patients with diabetes mellitus: integration of artificial intelligence-based drug prediction and clinical corroboration
Source: Front Pharmacol. 2023 May 18;14:1181711. doi: 10.3389/fphar.2023.1181711 (PMC10232753; doi:10.3389/fphar.2023.1181711)
Supplement: Supplementary file 1 [file DataSheet1.PDF]

# Supporting Information

1. **Table S1.** Statistics of nodes and interactions in the knowledge graph.

2. **Table S2.** Top 10-ranked drug candidates associated with the input of cataract-related genes.

3. **Table S3.** Table Covariates and their standardized names, codes, and data types used in the TriNeX database.

4. **Table S4.** Characteristics of patients prescribed Aspirin in T2DM patient group.

5. **Table S5.** Characteristics of patients prescribed Aspirin in hyperglycemia patient group.

6. **Table S6.** Characteristics of patients prescribed Acetylcysteine in T2DM patient group.

7. **Table S7.** Characteristics of patients prescribed Acetylcysteine in hyperglycemia patient group.

8. **Table S8.** Characteristics of patients prescribed Melatonin in T1DM patient group.

9. **Table S9.** Characteristics of patients prescribed Melatonin in T2DM patient group.

10. **Table S10.** Characteristics of patients prescribed Melatonin in hyperglycemia patient group.

11. **Table S11.** Characteristics of patients prescribed Ibuprofen in T1DM patient group.

12. **Table S12.** Characteristics of patients prescribed Ibuprofen in T2DM patient group.

13. **Table S13.** Characteristics of patients prescribed Ibuprofen in hyperglycemia patient group.

**1. Table S1.** Statistics of nodes and interactions in the knowledge graph

| Knowledge Type | Data Source | Interaction Type | Interaction Number | Node Type | Node Number |
|---|---|---|---|---|---|
| Phenome-level knowledge | Phenomebrowser Database | Drug-Mammalian Phenotype | 36,422 | Drug | 1,228 |
| | | | | Mammalian Phenotype | 1,363 |
| | | Drug-Human Phenotype Ontology | 175,713 | Drug | 1,429 |
| | | | | Human Phenotype Ontology | 3,003 |
| | Mouse Genome Informatics (MGI) Database | Gene-Mammalian Phenotype | 187,304 | Gene | 12,219 |
| | | | | Mammalian Phenotype | 9,916 |
| | Gene Ontology Annotation (GOA) Database | Gene-Gene Ontology | 204,862 | Gene | 16,283 |
| | | | | Gene Ontology | 15,924 |
| | Genotype-Tissue Expression (GTEx) database | Gene-Uberon Anatomy Ontology | 539,845 | Gene | 16,579 |
| | | | | Uberon Anatomy Ontology | 51 |
| | Human Phenotype Ontology (HPO) database | Disease-Human Phenotype Ontology | 87,154 | Disease | 7,172 |
| | | | | Human Phenotype Ontology | 6,784 |
| Genome-level knowledge | DrugBank database | Drug-Gene | 5,280 | Drug | 985 |
| | | | | Gene | 1,365 |
| | Mouse Genome Informatics (MGI) Database | Gene-Disease | 7,382 | Gene | 3,363 |
| | | | | Disease | 4,350 |
| Text-mined knowledge | TreatKB | Drug-Disease | 69,113 | Drug | 1,971 |
| | | | | Disease | 10,744 |

**2. Table S2.** Top 10-ranked drug candidates associated with the input of cataract-related genes

| Rank | Drug | Indication | Evidence |
|---|---|---|---|
| 1 | Aspirin | Pain, Inflammation, Rheumat oid arthritis | PMID_2101031, PMID_10201579 |
| 2 | Indomethacin | Rheumatoid arthritis | PMID_16138003, PMID_8675396 |
| 3 | Acetylcysteine | Dry eye syndrome, Respiratory diseases | PMID_34339721, PMID_28446133 |
| 4 | Theophylline | Asthma | |
| 5 | Melatonin | Sleep disorders | PMID_30074278, PMID_27365552 |
| 6 | Thalidomide | Myeloma | |
| 7 | Ibuprofen | Pain | PMID_2311679, PMID_12009421 |
| 8 | Ephedrine | Hypotension | |
| 9 | Tamoxifen | Breast cancer | |
| 10 | Pentoxifylline | Intermittent claudication | |

Note: PMID**: evidence from PubMed

**3. Table S3.** Table Covariates and their standardized names, codes, and data types used in the TriNeX database.

| Covariate | Name (abbreviation or code) | Datatype |
|---|---|---|
| Age | Age at index (AI) | Continuous |
| Sex | Female (F) | Present/absent |
| | Male (M) | Present/absent |
| Ethnicity | Hispanic/Latinx (2135-2) | Present/absent |
| | Not Hispanic/Latinx (2186-5) | Present/absent |
| Race | African American/Black (2054-5) | Present/absent |
| | White (2106-3) | Present/absent |
| | Asian (2028-9) | Present/absent |
| Comorbidities | Hypertensive diseases (I10-I16) | Present/absent |
| | DM mellitus (E08-E13) | Present/absent |
| | Tabaco use (Z72.0) | Present/absent |
| | Glaucoma (H40-H42) | Present/absent |
| | Dry eye syndrome (H04.12) | Present/absent |
| | Degeneration of macula and posterior pole (H35.3) | Present/absent |
| Other drug use | Drugs used in DM (A10) | Present/absent |

**4. Table S4.** Characteristics of patients prescribed Aspirin in T2DM patient group.

| Characteristics | Before Matching | | | After Matching | | |
|---|---|---|---|---|---|---|
| | Aspirin Cohort | Non-Aspirin Cohort | SMD | Aspirin Cohort | Non-Aspirin Cohort | SMD |
| Total No. | 88,697 | 184,164 | | 67,128 | 67,128 | |
| Age | 67.4 ± 10.7 | 64.4 ± 11.2 | 0.27* | 66.8 ± 10.6 | 66.4 ± 10.6 | 0.03 |
| Sex, % | | | | | | |
| Female | 54.9 | 58.1 | 0.06 | 55.1 | 55.1 | 0.0008 |
| Male | 45.1 | 41.8 | 0.06 | 44.9 | 44.9 | 0.0008 |
| Ethnicity, % | | | | | | |
| Hispanic/Latinx | 11.6 | 12.8 | 0.03 | 11.6 | 11.3 | 0.007 |
| Not Hispanic/Latinx | 66.6 | 56.9 | 0.21 | 65.7 | 67.2 | 0.03 |
| Race, % | | | | | | |
| African American/ Black | 25.1 | 20.8 | 0.11* | 24.3 | 25.2 | 0.021 |
| White | 58.4 | 55.1 | 0.06 | 58.5 | 58.4 | 0.001 |
| Asian | 2.5 | 3.2 | 0.03 | 2.6 | 2.6 | 0.001 |
| Comorbidities, % | | | | | | |
| Hypertension | 34.9 | 16.9 | 0.41* | 29.1 | 28.9 | 0.004 |
| Tobacco use | 7.3 | 1.7 | 0.27* | 3.5 | 3.6 | 0.003 |
| Glaucoma | 0.7 | 0.4 | 0.03 | 0.6 | 0.6 | 0.006 |
| Dry eye syndrome | 3.7 | 0.9 | 0.18* | 1.8 | 1.8 | 0.003 |
| Degeneration of macula and posterior pole | 3.4 | 0.4 | 0.21* | 0.9 | 1.0 | 0.004 |
| Other Drugs, % | | | | | | |
| Drugs used in DM | 21.9 | 9.3 | 0.35* | 17.1 | 17.2 | 0.007 |

Note: SMD - standardized mean differences. *SMD greater than 0.1, a threshold being recommended for declaring imbalance.

**5. Table S5.** Characteristics of patients prescribed Aspirin in hyperglycemia patient group.

| Characteristics | Before Matching | | | After Matching | | |
|---|---|---|---|---|---|---|
| | Aspirin Cohort | Non-Aspirin Cohort | SMD | Aspirin Cohort | Non-Aspirin Cohort | SMD |
| Total No. | 28,264 | 105,808 | | 22,784 | 22,784 | |
| Age | 68.5 ± 10.4 | 65.3 ± 10.4 | 0.31* | 68.2 ± 10.3 | 68.2 ± 10.3 | 0.0006 |
| Sex, % | | | | | | |
| Female | 57.4 | 62.1 | 0.09 | 57.6 | 58.3 | 0.01 |
| Male | 42.5 | 37.9 | 0.09 | 42.3 | 41.6 | 0.01 |
| Ethnicity, % | | | | | | |
| Hispanic/Latinx | 7.6 | 9.1 | 0.05 | 7.6 | 7.4 | 0.008 |
| Not Hispanic/Latinx | 73.2 | 65.7 | 0.16* | 72.6 | 72.4 | 0.003 |
| Race, % | | | | | | |
| African American/ Black | 18.1 | 15.8 | 0.05 | 17.8 | 17.4 | 0.01 |
| White | 68.5 | 63.1 | 0.11* | 68.3 | 68.8 | 0.01 |
| Asian | 2.4 | 3.5 | 0.06 | 2.5 | 2.3 | 0.01 |
| Comorbidities, % | | | | | | |
| Hypertension | 83.1 | 68.4 | 0.34* | 82.1 | 82.1 | 0.001 |
| Tobacco use | 6.6 | 5.1 | 0.06 | 6.4 | 6.2 | 0.009 |
| Glaucoma | 22.5 | 9.7 | 0.35* | 19.9 | 21.2 | 0.03 |
| Dry eye syndrome | 21.1 | 7.3 | 0.41* | 18.1 | 17.8 | 0.006 |
| Degeneration of macula and posterior pole | 14.1 | 4.1 | 0.35* | 11.4 | 11.2 | 0.004 |
| Other Drugs, % | | | | | | |
| Drugs used in DM | 36.5 | 22.7 | 0.31* | 35.1 | 34.6 | 0.008 |

Note: SMD - standardized mean differences. *SMD greater than 0.1, a threshold being recommended for declaring imbalance.

**6. Table S6.** Characteristics of patients prescribed Acetylcysteine in T2DM patient group.

| Characteristics | Before Matching | | | After Matching | | |
|---|---|---|---|---|---|---|
| | Acetylcysteine Cohort | Non-Acetylcysteine Cohort | SMD | Acetylcysteine Cohort | Non-Acetylcysteine Cohort | SMD |
| Total No. | 3,683 | 15,448 | | 3,683 | 3,683 | |
| Age | 68.7 ± 10.1 | 66.8 ± 10.5 | 0.18* | 68.7 ± 10.1 | 68.8 ± 9.94 | 0.01 |
| Sex, % | | | | | | |
| Female | 50.3 | 61.5 | 0.22* | 50.3 | 52.1 | 0.03 |
| Male | 49.6 | 38.4 | 0.22* | 49.6 | 47.9 | 0.03 |
| Ethnicity, % | | | | | | |
| Hispanic/Latinx | 6.7 | 13.6 | 0.22* | 6.7 | 6.5 | 0.007 |
| Not Hispanic/Latinx | 74.6 | 70.1 | 0.11* | 74.6 | 75.8 | 0.02 |
| Race, % | | | | | | |
| African American/ Black | 16.8 | 26.9 | 0.24* | 16.8 | 17.1 | 0.005 |
| White | 68.3 | 50.9 | 0.35* | 68.3 | 68.7 | 0.009 |
| Asian | 1.3 | 3.7 | 0.15* | 1.3 | 1.1 | 0.01 |
| Comorbidities, % | | | | | | |
| Hypertension | 57.1 | 48.1 | 0.17* | 57.1 | 56.2 | 0.01 |
| Tobacco use | 1.7 | 1.9 | 0.01 | 1.7 | 1.4 | 0.02 |
| Glaucoma | 10.9 | 11.1 | 0.007 | 10.9 | 9.9 | 0.03 |
| Dry eye syndrome | 8.2 | 11.1 | 0.09 | 8.2 | 7.5 | 0.02 |
| Degeneration of macula and posterior pole | 6.1 | 5.3 | 0.03 | 6.1 | 4.4 | 0.07 |
| Other Drugs, % | | | | | | |
| Drugs used in DM | 43.5 | 39.2 | 0.08 | 43.5 | 43.3 | 0.003 |

Note: SMD - standardized mean differences. *SMD greater than 0.1, a threshold being recommended for declaring imbalance.

**7. Table S7.** Characteristics of patients prescribed Acetylcysteine in hyperglycemia patient group.

| Characteristics | Before Matching | | | After Matching | | |
|---|---|---|---|---|---|---|
| | Acetylcysteine Cohort | Non-Acetylcysteine Cohort | SMD | Acetylcysteine Cohort | Non-Acetylcysteine Cohort | SMD |
| Total No. | 1,321 | 6,848 | | 1,321 | 1,321 | |
| Age | 69.5 ± 10.3 | 67.1 ± 10.5 | 0.22* | 69.5 ± 10.3 | 69.5 ± 9.73 | 0.004 |
| Sex, % | | | | | | |
| Female | 50.1 | 65.2 | 0.31* | 50.1 | 50.4 | 0.007 |
| Male | 49.9 | 34.7 | 0.31* | 49.9 | 49.5 | 0.007 |
| Ethnicity, % | | | | | | |
| Hispanic/Latinx | 4.3 | 11.1 | 0.25* | 4.3 | 3.1 | 0.07 |
| Not Hispanic/Latinx | 83.1 | 74.5 | 0.21* | 83.1 | 85.2 | 0.05 |
| Race, % | | | | | | |
| African American/ Black | 11.9 | 21.1 | 0.25* | 11.9 | 12.4 | 0.01 |
| White | 78.5 | 60.3 | 0.41* | 78.5 | 79.1 | 0.01 |
| Asian | 1.2 | 3.9 | 0.17* | 1.2 | 0.8 | 0.03 |
| Comorbidities, % | | | | | | |
| Hypertension | 93.1 | 83.3 | 0.31* | 93.1 | 93.9 | 0.03 |
| Tobacco use | 11.9 | 10.1 | 0.06 | 11.9 | 11.9 | 0 |
| Glaucoma | 25.8 | 27.1 | 0.02 | 25.8 | 24.5 | 0.02 |
| Dry eye syndrome | 32.9 | 56.5 | 0.48* | 32.9 | 35.5 | 0.05 |
| Degeneration of macula and posterior pole | 16.2 | 15.5 | 0.01 | 16.2 | 15.8 | 0.01 |
| Other Drugs, % | | | | | | |
| Drugs used in DM | 67.1 | 46.4 | 0.42* | 67.1 | 66.9 | 0.001 |

Note: SMD - standardized mean differences. *SMD greater than 0.1, a threshold being recommended for declaring imbalance.

**8. Table S8.** Characteristics of patients prescribed Melatonin in T1DM patient group.

| Characteristics | Before Matching | | | After Matching | | |
|---|---|---|---|---|---|---|
| | Melatonin Cohort | Non-Melatonin Cohort | SMD | Melatonin Cohort | Non-Melatonin Cohort | SMD |
| Total No. | 6,875 | 27,555 | | 4,511 | 4,511 | |
| Age | 65.8 ± 12 | 61.4 ± 11.2 | 0.37* | 64.8 ± 11.9 | 64.6 ± 11 | 0.01 |
| Sex, % | | | | | | |
| Female | 52.5 | 51.7 | 0.01 | 51.8 | 52.1 | 0.003 |
| Male | 47.4 | 48.2 | 0.01 | 48.1 | 47.9 | 0.003 |
| Ethnicity, % | | | | | | |
| Hispanic/Latinx | 9.2 | 9.3 | 0.004 | 9.1 | 9.2 | 0.003 |
| Not Hispanic/ Latinx | 72.6 | 64.5 | 0.17* | 71.8 | 72.3 | 0.009 |
| Race, % | | | | | | |
| African American/Black | 25.4 | 20.6 | 0.11* | 24.6 | 24.8 | 0.003 |
| White | 58.9 | 60.5 | 0.03 | 59.7 | 59.3 | 0.008 |
| Asian | 1.1 | 1.1 | 0.01 | 1.1 | 1.1 | 0.001 |
| Comorbidities, % | | | | | | |
| Hypertension | 97.5 | 88.1 | 0.37* | 97.2 | 97.3 | 0.008 |
| Tobacco use | 11.3 | 5.1 | 0.22* | 10.3 | 10.1 | 0.008 |
| Glaucoma | 30.9 | 13.7 | 0.42* | 26.4 | 27.1 | 0.01 |
| Dry eye syndrome | 27.8 | 6.8 | 0.57* | 20.2 | 20.2 | 0.002 |
| Degeneration of macula and posterior pole | 22.5 | 5.7 | 0.49* | 16.6 | 17.1 | 0.01 |
| Other Drugs, % | | | | | | |
| Drugs used in DM | 96.7 | 77.9 | 0.58* | 96.2 | 96.1 | 0.01 |
| Caffeine | 11.9 | 7.1 | 0.17* | 11.1 | 10.7 | 0.01 |

Note: SMD - standardized mean differences. *SMD greater than 0.1, a threshold being recommended for declaring imbalance.

**9. Table S9.** Characteristics of patients prescribed Melatonin in T2DM patient group.

| Characteristics | Before Matching | | | After Matching | | |
|---|---|---|---|---|---|---|
| | Melatonin Cohort | Non-Melatonin Cohort | SMD | Melatonin Cohort | Non-Melatonin Cohort | SMD |
| Total No. | 32,033 | 155,804 | | 21,803 | 21,803 | |
| Age | 68.9 ± 10.7 | 64 ± 10.1 | 0.47* | 67.8 ± 10.5 | 67.4 ± 10.3 | 0.03 |
| Sex, % | | | | | | |
| Female | 53.8 | 53.1 | 0.01 | 53.1 | 52.5 | 0.008 |
| Male | 46.1 | 46.9 | 0.01 | 46.9 | 47.4 | 0.008 |
| Ethnicity, % | | | | | | |
| Hispanic/Latinx | 9.6 | 9.1 | 0.01 | 9.4 | 9.5 | 0.002 |
| Not Hispanic/ Latinx | 72.1 | 65.3 | 0.14* | 71.1 | 72.3 | 0.02 |
| Race, % | | | | | | |
| African American/Black | 22.7 | 20.6 | 0.04 | 21.8 | 21.7 | 0.002 |
| White | 60.6 | 59.8 | 0.01 | 61.1 | 61.5 | 0.007 |
| Asian | 1.8 | 1.9 | 0.009 | 1.8 | 1.9 | 0.002 |
| Comorbidities, % | | | | | | |
| Hypertension | 63.2 | 28.1 | 0.75* | 58.3 | 58.1 | 0.003 |
| Tobacco use | 2.3 | 0.7 | 0.12* | 2.1 | 2.1 | 0.001 |
| Glaucoma | 13.5 | 2.6 | 0.41* | 8.2 | 8.7 | 0.01 |
| Dry eye syndrome | 8.8 | 1.3 | 0.34* | 4.4 | 4.8 | 0.02 |
| Degeneration of macula and posterior pole | 7.1 | 0.6 | 0.34* | 2.5 | 2.8 | 0.01 |
| Other Drugs, % | | | | | | |
| Drugs used in DM | 55.3 | 18.2 | 0.83* | 49.7 | 50.1 | 0.006 |
| Caffeine | 5.6 | 3.1 | 0.12* | 5.1 | 4.7 | 0.01 |

Note: SMD - standardized mean differences. *SMD greater than 0.1, a threshold being recommended for declaring imbalance.

**10. Table S10.** Characteristics of patients prescribed Melatonin in hyperglycemia patient group.

| Characteristics | Before Matching | | | After Matching | | |
|---|---|---|---|---|---|---|
| | Melatonin Cohort | Non-Melatonin Cohort | SMD | Melatonin Cohort | Non-Melatonin Cohort | SMD |
| Total No. | 14,513 | 88,323 | | 11,297 | 11,297 | |
| Age | 69.4 ± 10.8 | 64.9 ± 9.66 | 0.43* | 68.8 ± 10.8 | 68.7 ± 10.3 | 0.01 |
| Sex, % | | | | | | |
| Female | 56.4 | 56.5 | 0.002 | 56.3 | 57.1 | 0.01 |
| Male | 43.5 | 43.4 | 0.002 | 43.6 | 42.8 | 0.01 |
| Ethnicity, % | | | | | | |
| Hispanic/Latinx | 6.7 | 6.7 | 0.0009 | 6.7 | 6.2 | 0.01 |
| Not Hispanic/ Latinx | 75.6 | 72.2 | 0.07 | 75.1 | 75.3 | 0.004 |
| Race, % | | | | | | |
| African American/Black | 16.4 | 15.3 | 0.02 | 16.4 | 17.1 | 0.01 |
| White | 69.8 | 68.1 | 0.03 | 69.6 | 69.1 | 0.01 |
| Asian | 1.5 | 2.2 | 0.04 | 1.6 | 1.5 | 0.006 |
| Comorbidities, % | | | | | | |
| Hypertension | 90.1 | 79.1 | 0.31* | 89.6 | 89.7 | 0.005 |
| Tobacco use | 10.6 | 6.2 | 0.16* | 10.3 | 10.1 | 0.006 |
| Glaucoma | 24.6 | 11.1 | 0.36* | 22.5 | 23.7 | 0.02 |
| Dry eye syndrome | 28.6 | 8.8 | 0.52* | 25.1 | 25.1 | 0.002 |
| Degeneration of macula and posterior pole | 17.3 | 4.4 | 0.42* | 14.1 | 14.3 | 0.004 |
| Other Drugs, % | | | | | | |
| Drugs used in DM | 54.5 | 34.4 | 0.41* | 53.1 | 52.7 | 0.006 |
| Caffeine | 13.6 | 9.7 | 0.12* | 13.1 | 12.1 | 0.02 |

Note: SMD - standardized mean differences. *SMD greater than 0.1, a threshold being recommended for declaring imbalance.

**11. Table S11.** Characteristics of patients prescribed Ibuprofen in T1DM patient group.

| Characteristics | Before Matching | | | After Matching | | |
|---|---|---|---|---|---|---|
| | Ibuprofen Cohort | Non-Ibuprofen Cohort | SMD | Ibuprofen Cohort | Non-Ibuprofen Cohort | SMD |
| Total No. | 6,891 | 5,985 | | 5,314 | 5,314 | |
| Age | 61.7 ± 12.2 | 65 ± 11.3 | 0.28* | 63.9 ± 11.3 | 63.9 ± 11.2 | 0.001 |
| Sex, % | | | | | | |
| Female | 56.6 | 52.1 | 0.09 | 53.5 | 53.3 | 0.003 |
| Male | 43.3 | 47.8 | 0.09 | 46.4 | 46.6 | 0.003 |
| Ethnicity, % | | | | | | |
| Hispanic/Latinx | 12.2 | 9.4 | 0.08 | 9.9 | 10.2 | 0.009 |
| Not Hispanic/Latinx | 66.5 | 65.5 | 0.02 | 67.1 | 66.4 | 0.01 |
| Race, % | | | | | | |
| African American/Black | 27.8 | 23.1 | 0.11* | 24.4 | 24.7 | 0.006 |
| White | 53.9 | 61.9 | 0.16* | 59.8 | 59.3 | 0.01 |
| Asian | 1.7 | 1.5 | 0.01 | 1.5 | 1.5 | 0.004 |
| Comorbidities, % | | | | | | |
| Hypertension | 89.3 | 90.6 | 0.04 | 90.6 | 90.5 | 0.005 |
| Tobacco use | 5.6 | 4.7 | 0.04 | 5.1 | 5.1 | 0.0008 |
| Glaucoma | 25.8 | 26.1 | 0.004 | 26.1 | 26.3 | 0.005 |
| Dry eye syndrome | 17.5 | 15.7 | 0.04 | 16.2 | 16.4 | 0.005 |
| Degeneration of macula and posterior pole | 12.8 | 15.1 | 0.06 | 14.1 | 13.9 | 0.003 |
| Other Drugs, % | | | | | | |
| Drugs used in DM | 92.1 | 87.5 | 0.15* | 90.4 | 90.1 | 0.01 |

Note: SMD - standardized mean differences. *SMD greater than 0.1, a threshold being recommended for declaring imbalance.

**12. Table S12.** Characteristics of patients prescribed Ibuprofen in T2DM patient group.

| Characteristics | Before Matching | | | After Matching | | |
|---|---|---|---|---|---|---|
| | Ibuprofen Cohort | Non-Ibuprofen Cohort | SMD | Ibuprofen Cohort | Non-Ibuprofen Cohort | SMD |
| Total No. | 42,298 | 40,837 | | 34,090 | 34,090 | |
| Age | 64.6 ± 10.6 | 68 ± 9.96 | 0.33* | 66.6 ± 9.93 | 66.6 ± 9.81 | 0.002 |
| Sex, % | | | | | | |
| Female | 59.7 | 56.4 | 0.06 | 57.8 | 58.2 | 0.008 |
| Male | 40.2 | 43.5 | 0.06 | 42.1 | 41.7 | 0.008 |
| Ethnicity, % | | | | | | |
| Hispanic/Latinx | 15.1 | 10.1 | 0.15* | 11.7 | 11.8 | 0.003 |
| Not Hispanic/Latinx | 64.5 | 65.3 | 0.01 | 65.7 | 65.4 | 0.004 |
| Race, % | | | | | | |
| African American/Black | 28.4 | 23.3 | 0.11* | 25.1 | 25.8 | 0.01 |
| White | 51.7 | 58.4 | 0.13* | 55.7 | 55.2 | 0.008 |
| Asian | 2.9 | 3.3 | 0.02 | 3.2 | 3.2 | 0.002 |
| Comorbidities, % | | | | | | |
| Hypertension | 40.8 | 36.9 | 0.08 | 39.7 | 39.1 | 0.01 |
| Tobacco use | 0.7 | 0.5 | 0.02 | 0.6 | 0.6 | 0.008 |
| Glaucoma | 7.7 | 7.1 | 0.02 | 7.1 | 7.5 | 0.01 |
| Dry eye syndrome | 4.1 | 3.5 | 0.02 | 3.6 | 3.8 | 0.01 |
| Degeneration of macula and posterior pole | 2.8 | 2.7 | 0.002 | 2.7 | 2.8 | 0.005 |
| Other Drugs, % | | | | | | |
| Drugs used in DM | 30.5 | 24.8 | 0.12* | 28.1 | 27.5 | 0.01 |

Note: SMD - standardized mean differences. *SMD greater than 0.1, a threshold being recommended for declaring imbalance.

**13. Table S13.** Characteristics of patients prescribed Ibuprofen in hyperglycemia patient group.

| Characteristics | Before Matching | | | After Matching | | |
|---|---|---|---|---|---|---|
| | Ibuprofen Cohort | Non-Ibuprofen Cohort | SMD | Ibuprofen Cohort | Non-Ibuprofen Cohort | SMD |
| Total No. | 17,266 | 16,478 | | 14,247 | 14,247 | |
| Age | 66.2 ± 10.2 | 69.3 ± 9.51 | 0.31* | 68.1 ± 9.45 | 68.2 ± 9.46 | 0.01 |
| Sex, % | | | | | | |
| Female | 60.3 | 57.5 | 0.05 | 58.8 | 58.8 | 0.001 |
| Male | 39.6 | 42.4 | 0.05 | 41.1 | 41.1 | 0.001 |
| Ethnicity, % | | | | | | |
| Hispanic/Latinx | 10.5 | 6.3 | 0.15* | 6.9 | 7.2 | 0.01 |
| Not Hispanic/Latinx | 69.9 | 73.1 | 0.06 | 72.4 | 72.2 | 0.003 |
| Race, % | | | | | | |
| African American/Black | 20.6 | 14.6 | 0.15* | 15.9 | 16.5 | 0.01 |
| White | 62.1 | 70.6 | 0.18* | 68.5 | 67.7 | 0.01 |
| Asian | 3.2 | 3.5 | 0.01 | 3.5 | 3.5 | 0.001 |
| Comorbidities, % | | | | | | |
| Hypertension | 79.4 | 80.2 | 0.01 | 79.9 | 79.9 | 0.0001 |
| Tobacco use | 6.8 | 4.4 | 0.11* | 4.7 | 5.1 | 0.01 |
| Glaucoma | 22.3 | 20.7 | 0.03 | 21.1 | 21.3 | 0.009 |
| Dry eye syndrome | 22.1 | 18.9 | 0.07 | 20.1 | 19.9 | 0.0007 |
| Degeneration of macula and posterior pole | 11.2 | 12.4 | 0.03 | 11.6 | 12.2 | 0.01 |
| Other Drugs, % | | | | | | |
| Drugs used in DM | 40.2 | 33.1 | 0.14* | 36.3 | 35.8 | 0.01 |

Note: SMD - standardized mean differences. *SMD greater than 0.1, a threshold being recommended for declaring imbalance.
